# Azelaic Acid-Mediated Resistance in Rice Against Infection by *Bipolaris oryzae*

**DOI:** 10.3390/plants15040567

**Published:** 2026-02-11

**Authors:** Geovane Souza Gudin, Leandro Castro Silva, Bárbara Bezerra Menezes Picanço, Aline Vieira Barros, Verônica Vieira Brás, Fabrício Ávila Rodrigues

**Affiliations:** Laboratório da Interação Planta-Patógeno, Departamento de Fitopatologia, Universidade Federal de Viçosa, Viçosa 36570-900, Minas Gerais, Brazil; geovane.gudin@ufv.br (G.S.G.); leandrocsilva1989@gmail.com (L.C.S.); barbara.picanco@gmail.com (B.B.M.P.); alinevieiradebarros@gmail.com (A.V.B.); brasveronica@gmail.com (V.V.B.)

**Keywords:** *Oryza sativa*, antioxidative metabolism, defense-related enzymes, induced resistance, plant defense reactions

## Abstract

Brown spot, caused by the fungus *Bipolaris oryzae*, has led to significant yield losses in rice production worldwide. This study hypothesized that azelaic acid (AzA) could reduce brown spot symptoms in rice leaves by potentiating biochemical defense reactions. A 2 × 2 factorial experiment was arranged in a completely randomized design with five replications per sampling time. The factors studied were plants sprayed with water (control) or AzA (10 mM; 7.5 mL per plant), either non-inoculated or inoculated with *B. oryzae*. In the in vitro assay, conidia exposed to AzA solutions at rates of 2.5, 5, 7.5, and 10 mM and to the fungicide solution did not form germ tubes compared to those in the control (water) treatment. The area of fungal colonies on oat–agar medium was reduced for the fungicide and AzA (rates increasing from 2.5 to 10 mM) treatments compared to the control (water) treatment. The EC_50_ value was 3.8 mM AzA. Brown spot severity significantly decreased by 57, 48, 52, and 58% at 36, 60, 84, and 108 h after inoculation (hai) for AzA-sprayed plants compared to water-sprayed ones. The area under brown spot progress curve significantly decreased by 53% for AzA-sprayed plants compared to water-sprayed ones. Greatest activities of defense-related enzymes (chitinase at 108 hai, *β*-1,3-glucanase at 60 hai, phenylalanine ammonia-lyase at 60 and 108 hai, and lipoxygenase at 84 and 108 hai), a higher concentration of lignin at 84 and 108 hai, and a more robust antioxidative metabolism (higher activities of ascorbate peroxidase at 36 hai, catalase at 84 and 108 hai, and superoxide dismutase at 84 hai) were obtained for AzA-sprayed infected plants. The higher concentration of the superoxide anion radical in AzA-sprayed infected leaves helped to intensify the cell defense reactions against fungal infection and had a fungistatic effect against its hyphae and conidia germination. The findings of this study provide valuable insights into using AzA to potentiate foliar defense reactions in rice plants to hamper the infection by *B. oryzae*.

## 1. Introduction

It is well known that rice (*Oryza sativa* L.) plays a pivotal role in food security and social stability worldwide [1]. In rice fields across countries in Asia, Africa, and Latin America, the occurrence of epidemics of brown spot, caused by the necrotrophic fungus *Bipolaris oryzae* (Breda de Haan) Shoemaker (teleomorph *Cochliobolus miyabeanus* (Iot & Kuribayashi) Drechs ex Dastur), has caused extensive yield losses and reduced grain quality [2]. On infected leaf blades, light reddish-brown lesions appear, with a gray center, a dark to reddish-brown margin, and a bright yellow halo as they expand [2]. The management of brown spot in rice-growing regions has been achieved by using cultivars with higher levels of partial resistance, fungicide spray, avoiding conditions for drought stress, balanced plant nutrition (adequate foliar contents of calcium, manganese, magnesium, potassium, and iron), and silicon supply [2,3].

The adoption of more sustainable agricultural practices to address sustainable development goals (e.g., poverty reduction, food security, reduced impact on different biomes worldwide, and climate resilience) is an urgent demand of our society. In this scenario, more environmentally friendly strategies for brown spot control need to be made available for rice growers. Plants can exhibit greater resilience to disease epidemics when exposed to different types of inducers of resistance that boost their defense mechanisms [4]. The activation of systemic acquired resistance (SAR) is one strategy that plants use to cope with pathogen infection [5]. SAR is mainly regulated by the hormone salicylic acid (SA) with the co-participation of azelaic acid (AzA). AzA is C9 dicarboxylic acid (oxylipin) found in the interconnected membrane systems of the endoplasmic reticulum, plasmodesmata, and thylakoids [6,7]. This oxylipin originates from the non-enzymatic and enzymatic hydrolysis of C18 fatty acids [e.g., oleic acid (18:1) or their unsaturated derivatives, linoleic acid (18:2) and linolenic acid (18:3)] [6,7]. The pool of AzA precursors can increase due to an abundant pool of free unsaturated fatty acids originating from oxidative stress in infected plant tissues [8]. At the cytosol, the interaction of glycerol-3-phosphate with two lipid transfer proteins [defective in induced resistance 1 (DIR1) and azelaic acid-induced 1 (AZI1)] highlights the important contribution of AzA for the SAR signaling pathway [9]. It has been shown that an exogenous supply of AzA in certain crops enhances their defense responses against infection by various pathogens [9,10,11,12,13]. For instance, the infection process of the biotrophic fungus *Phakopsora pachyrhizi* on soybean leaves sprayed with AzA was profoundly hampered, as evidenced by fewer necrotic lesions containing fewer uredinia [14]. According to the authors, the leaf tissues responded more promptly by activating a range of defense-related genes (*PAL1.1*, *PAL1.3*, *PR1-A*, *PR10*, *MMP2*, *CHIB1*, *ACS*, *NAC19*, and *URE*) in response to infection by *P. pachyrhizi* [14]. Coffee plants sprayed with AzA displayed less sporulation of *Hemileia vastatrix* [15]. According to the authors, the area under coffee leaf rust progress curve and the intensity of fungal sporulation were significantly lower by 82% and 83%, respectively, and the incubation period was higher by 31% for AzA-sprayed plants compared to water-sprayed ones [15]. The literature reports the fungitoxic effect of AzA against the germination of spores of different fungal species (e.g., *P. pachyrhizi* and *H. vastatrix*) due to its ability to disrupt cellular membranes, inhibit key enzymes involved in fungal metabolism, induce oxidative stress, and interfere with fungal cell wall synthesis and function that will lower fungal growth [9,10,11,12,13,14,15].

Considering the social and economic importance of rice worldwide, the present study hypothesized that AzA could potentiate cellular defense reactions in rice leaves to hinder the infection process by *B. oryzae*. This hypothesis was tested by performing a series of biochemical analyses (activities of defense-related and antioxidant enzymes, phenolic pool, lignin, and reactive oxygen species) on rice plants that were either non-challenged or challenged with *B. oryzae* and previously non-sprayed or sprayed with AzA.

## 2. Materials and Methods

### 2.1. In Vitro Assays

Oat–agar (OA) medium assay: A total of 15 mL of melted OA medium was separately mixed with different volumes of a stock solution of AzA (100 mM; CAS 123-99-9; Sigma-Aldrich, São Paulo, Brazil), prepared using Milli-Q water, to obtain the final concentrations of 2.5, 5, 7.5, and 10 mM. The OA medium amended with each concentration of AzA was poured into Petri plates. The control treatments corresponded to: (*i*) OA medium without AzA and (*ii*) OA medium amended with fungicide [7 mL/L, 0.41 M; Nativo^®^, tebuconazole (100 g/L) and trifloxystrobin (200 g/L); Bayer S.A., São Paulo, Brazil]. One plug of OA medium (0.5 mm^2^) containing fungal mycelia was obtained from the edge of a five-day-old colony of *B. oryzae* (isolate UFV-DFP *Bo*-12) using a sterile cork-borer. This plug was placed in the center of each Petri dish. Dishes were kept in a growth chamber (temperature of 25 °C and photoperiod of 12 h light/12 h dark). The Petri dishes containing the fungal colonies were scanned at 600 dpi resolution. The images were analyzed using the function measure of the Axion Vision software (version 4.8.1) to obtain the area (mm^2^) of each fungal colony. The area of the plug (0.5 mm^2^) was subtracted from the total area of each fungal colony. Mycelial growth inhibition, as a percentage, was calculated as follows: 100 − [(area of fungal colony per replication of each treatment/area of fungal colony per replication of the control treatment (AzA or fungicide) × 100]. The effective concentration (EC) of AzA capable of reducing fungal mycelia growth by 50% (EC_50_) was calculated using the interpolation method to obtain the best-fitted dose–response following the procedures of Weems and Bradley [16].

Glass slide assay: A total of 1 mL of a conidial suspension of *B. oryzae* (isolate UFV-DFP *Bo*-12; 5 × 10^3^ conidia/mL) was mixed with different volumes of a stock solution (40 mM) of AzA, prepared with Milli-Q water, to obtain final concentrations of 2.5, 5, 7.5, and 10 mM. A total of 30 µL of conidial suspension containing the different concentrations of AzA was transferred to a glass slide and covered with a coverslip. The control treatments corresponded to: (*i*) suspension of conidia without AzA and (*ii*) suspension of conidia mixed with fungicide [7 mL/L, 0.41 M; Nativo^®^, tebuconazole (100 g/L) and trifloxystrobin (200 g/L); Bayer S.A., Brazil]. The glass slides were kept in a growth chamber (temperature of 22 °C, in darkness) for 12 h. After this period, each glass slide received 20 μL of lacto-fuchsin solution [1:1 mixture of acid fuchsin (1 mg/mL) with lactic acid (85%)] to both stain and stop conidia germination (CG). One hundred conidia on each glass slide were randomly viewed under a light microscope (Carl Zeiss AxioImager A1, Oberkochen, Baden-Wurttemberg, Germany) using bright-field illumination at 100× magnification. The images of the CG details were acquired digitally (model AxioCam HR and Axion Vision software v. 4.8.1) at 400× magnification. A conidium with a germ tube larger than its diameter was considered germinated. The percentage of CG was calculated for each treatment replication.

### 2.2. Plant Growth

Rice seeds (cultivar Metica-1, susceptible to *B. oryzae*) were sown in each plastic pot (10 seeds per pot) containing 2 kg of substrate [17]. Four plants were left in each pot, which received (weekly) 100 mL of nutrient solution [17]. Plants were grown in a greenhouse [temperature of 25 ± 2 °C, 70 ± 5% relative humidity, and natural photosynthetically active radiation of 922 ± 20 μmol photons m^−2^ s^−1^ measured at midday] until being non-inoculated or inoculated with *B. oryzae*.

### 2.3. Production of Inoculum from B. oryzae and Plant Inoculation

The inoculum from *B. oryzae* (isolate UFV-DFP *Bo*-12) was produced according to Gudin et al. [17]. Plants at the V7 growth stage [18] were inoculated with a fungal conidial suspension (5 × 10^3^ conidia/mL; 8 mL per plant) using a VL Airbrush atomizer (Paasche Airbrush Co., Chicago, IL, USA) until runoff. For disease development, inoculated plants were kept in a growth chamber (temperature of 25 ± 2 °C and 90 ± 5% relative humidity) in the dark for 24 h. After this period, plants were transferred to a plastic mist growth chamber (MGC) inside a greenhouse for the duration of the experiment. The temperature inside the MGC ranged from 25 ± 2 °C (day) to 20 ± 2 °C (night). The relative humidity was maintained at 92 ± 3% using a misting system [17]. The maximum natural photon flux density at the plant canopy height was approximately 900 µmol m^−2^ s^−1^. Non-inoculated plants were kept in separate chambers but were exposed to the same conditions as the inoculated plants during the experiments.

### 2.4. Spraying Rice Plants with the Solution of AzA

Rice plants were sprayed with a solution of AzA (10 mM; 7.5 mL per plant), prepared using deionized water, 48 h before inoculation with *B. oryzae*. The water-sprayed plants served as the control treatment.

### 2.5. Evaluation of Brown Spot Severity

The sixth and seventh leaves, from base to top, of each plant per replication of each treatment were used to evaluate the brown spot severity at 36, 60, 84, and 108 h after inoculation (hai) using the diagrammatic scale proposed by Lenz et al. [19]. These time-points were selected to cover the infection process of *B. oryzae*, from conidium germination (36 hai) through fungal penetration and colonization of plant tissues (60–84 dai) to sporulation (108 dai) [3]. The trapezoidal integration of the disease progress curve for each leaf obtained from the plants of the replication of each treatment was used to obtain the values to calculate the area under brown spot progress curve (AUBSPC) [20].

### 2.6. Biochemical Analysis

The sixth and seventh leaves, from base to top, of each plant per replication of each treatment were collected at 36, 60, 84, and 108 hai using liquid nitrogen and immediately stored in a vertical ultrafreezer (−80 °C) until the biochemical analysis.

Determining the concentration of malondialdehyde (MDA): Frozen leaf tissue (0.1 g) was ground into a fine powder with liquid nitrogen in a mixer mill MM 400 (Retsch) with the addition of 1% polyvinylpyrrolidone (wt/vol). The fine powder was homogenized in 2 mL of 0.1% (wt/vol) trichloroacetic acid (TCA) solution. The homogenate was centrifuged at 12,000 g for 15 min at 4 °C. After centrifugation, 0.25 mL of the supernatant was added to 0.75 mL of the total 2-thiobarbituric acid solution (0.5% in 20% TCA) and kept in a thermomixer for 60 min at 99 °C (Eppendorf, Hamburg, Germany) [21]. The reaction was stopped in an ice bath. After the solution reached room temperature, the absorbance was read at 532 nm and subtracted from the non-specific absorbance at 600 nm. A standard sucrose curve was used to correct for interference from soluble sugars in the samples. The molar extinction coefficient of 155 mM^−1^ cm^−1^ was used to calculate the MDA concentration [21].

Determining the concentrations of hydrogen peroxide (H_2_O_2_) and superoxide anion radical (O_2_^•−^): Frozen leaf tissue (0.1 g) was ground as described above and the fine powder was homogenized in 2 mL of 0.1% (wt/vol) of TCA. The homogenate was centrifuged at 12.000 g for 15 min at 4 °C and an aliquot of the supernatant was reacted with a mixture containing potassium phosphate buffer (10 mM and pH 7.0) and potassium iodide solution and incubated for 5 min. Absorbance was determined at 390 nm. The H_2_O_2_ concentration was determined based on a standard curve made with known concentrations of H_2_O_2_ [22]. Frozen leaf tissue (0.1 g) was ground as described above and the fine powder was homogenized in 2 mL of a solution containing sodium phosphate buffer (100 mM and pH 7.2) and sodium diethyldithiocarbamate (SDD) (1 mM). The homogenate was centrifuged at 22.000 g for 20 min at 4 °C. After centrifugation, an aliquot of the supernatant was added to react with a solution containing sodium phosphate buffer (100 mM and pH 7.2), SDD (1 mM), and nitroblue tetrazolium (0.25 mM). The O_2_^•−^ concentration was determined by subtracting the absorbance of the final product from the initial absorbance at 540 nm [23].

Determining activities of defense-related enzymes: Frozen leaf tissue (0.1 g) was ground as described above and the fine powder was homogenized in 2 mL of a solution containing potassium phosphate buffer (50 mM and pH 6.8), EDTA (1 mM), phenylmethyl sulphonyl fluoride (PMSF) (1 mM), and polyvinylpolypyrrolidone (PVPP) (0.5% wt/vol). The homogenate was centrifuged at 12.000 g for 15 min at 4 °C and the supernatant was used to determine the activities of chitinase (CHI) (EC 3.2.1.14), *β*-1,3-glucanase (GLU) (EC 3.2.1.39), phenylalanine ammonia-lyase (PAL) (EC 4.3.1.5), polyphenoloxidase (PPO) (EC 1.10.3.1), and lipoxygenase (LOX) (EC 1.13.11.12) following the procedures described by Fortunato et al. [24].

Determining the activities of antioxidant enzymes: Frozen leaf tissue (0.1 g) was ground as described above and the fine powder was homogenized in 2 mL of potassium phosphate buffer (100 mM and pH 6.8) containing EDTA (0.1 mM), PMSF (1 mM), and PVPP (0.5% wt/vol). The homogenate was centrifuged at 13.000 g for 15 min at 4 °C and the supernatant was used as an extract to determine ascorbate peroxidase (APX, EC 1.11.1.11), catalase (CAT, EC 1.11.1.6), glutathione reductase (GR, EC 1.8.1.7), peroxidase (POX) (EC 1.11.1.7), and superoxide dismutase (SOD, EC 1.15.1.1) activities as previously described [25,26].

Determining the concentrations of total soluble phenolics (TSP) and lignin-thioglycolic acid (LTGA) derivatives: Frozen leaf tissue (0.1 g) was ground as described above and the fine powder was homogenized in 1 mL of 80% (*v*/*v*) methanol solution. The crude extract was shaken at 300 rpm at 25 °C for 12 h and the mixture was centrifuged at 13,000 g for 30 min. The TSP concentration was determined in the methanolic extract and the pellet was kept at 20 °C to determine the concentration of LTGA derivatives following the procedures of Fortunato et al. [24].

### 2.7. Experimental Design and Statistical Analysis of Data

For the in vitro assays, the experiments were arranged in a completely randomized design (CRD) with six treatments (two controls and four AzA concentrations) and ten replications. One Petri dish or one glass slide corresponded to the replications of each treatment. The experiment was repeated once. A 2 × 2 factorial experiment, consisting of plants sprayed with water (control) or AzA and non-inoculated or inoculated with *B. oryzae*, was arranged in a CRD with five replications per evaluation time to assess brown spot severity. Each experimental unit consisted of a plastic pot containing four plants. Leaf samples for the biochemical assays were obtained from another 2 × 2 factorial experiment using the same factors described above and five replications. These experiments were repeated once. Data from CG were not subjected to analysis of variance (ANOVA) considering that they did not germinate after being exposed to the AzA rates ranging from 2.5 to 10 mM and also to the fungicide. For other variables and parameters, data were subjected to ANOVA, and comparisons between control and AzA treatments as well as between non-inoculated and inoculated plants were performed using the *F* test (*p* ≤ 0.05). Data were checked for normality and homogeneity of variance before ANOVA. The ANOVA was obtained for each evaluation time. The procedures described by Moore and Dixon [27] were followed to combine the data from the variables and parameters evaluated from the repeated experiments. The Minitab Statistical software version 22.1 was used for the statistical analysis mentioned above [28]. Data from all variables and parameters obtained from control and AzA treatments for non-inoculated and inoculated plants at 108 hai were used for the principal component analysis (PCA) using the software R version 4.5.2 [29].

## 3. Results

### 3.1. Germination of Conidium and Fungal Hyphal Growth from B. oryzae Affected by AzA

**Figure 1:**
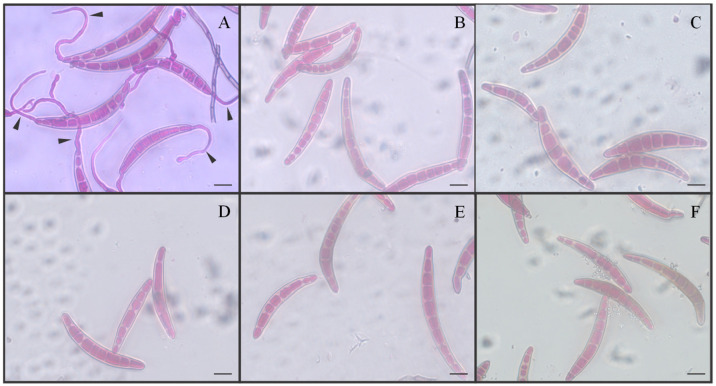
Germination of conidia from *Bipolaris oryzae* in vitro when exposed to different concentrations of azelaic acid. The rates of AzA tested were 2.5, 5, 7.5, and 10 mM, respectively, for (**B**–**E**). Conidia non-exposed to AzA (**A**) or exposed to fungicide (**F**) were the control treatments. Germ tubes (arrowheads). Scale bars = 5 μm.

**Figure 2:**
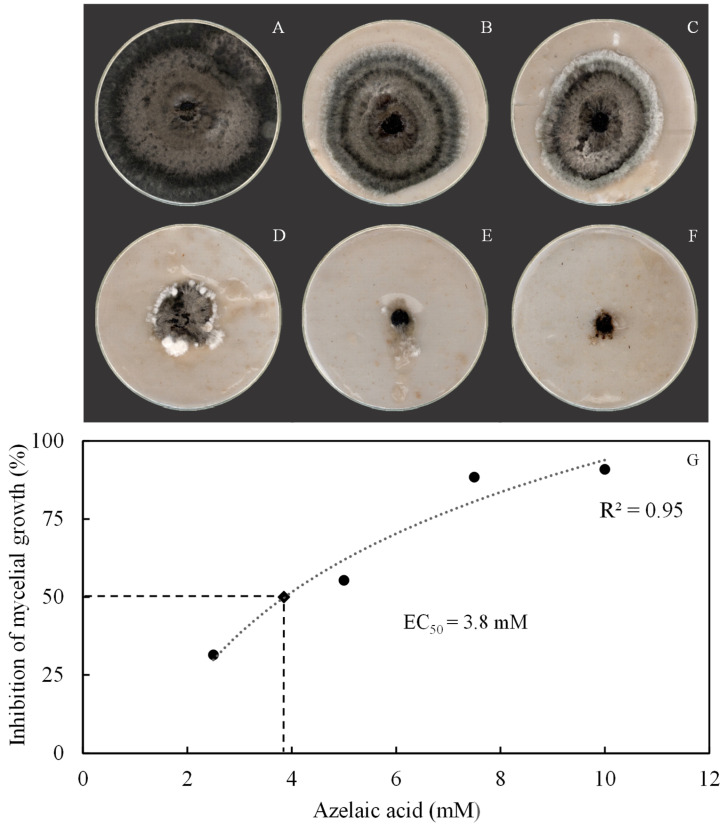
Aspect of growth of *Bipolaris oryzae* mycelia in Petri dishes containing oat–agar medium amended with azelaic acid concentrations of 2.5, 5, 7.5, and 10 mM, respectively, for (**B**–**E**). Fungal mycelia non-exposed to AzA (**A**) or exposed to fungicide (**F**) were the control treatments. Fungal mycelial growth was inhibited in 50% with an effective concentration (EC_50_) of 3.8 mM AzA (**G**).

The effect of AzA against conidium germination and fungal hyphal growth was determined during in vitro assays in an attempt to explain the effect of this molecule against fungal physiology. Interestingly, conidia did not form germ tubes in the presence of AzA at doses that ranged from 2.5 to 10 mM or in the presence of fungicide in contrast to those from the control treatment (Figure 1A–F). Poorly developed fungal colonies were formed on OA medium amended with AzA rates from 2.5 to 10 mM compared with the control treatment (Figure 2A–E). Fungal colonies were smaller after being in contact with fungicide (Figure 2F). The rate of 3.8 mM AzA corresponded to the EC_50_ value (Figure 2G).

### 3.2. Disease Symptoms and Severity, as Well as AUBSPC

**Figure 3:**
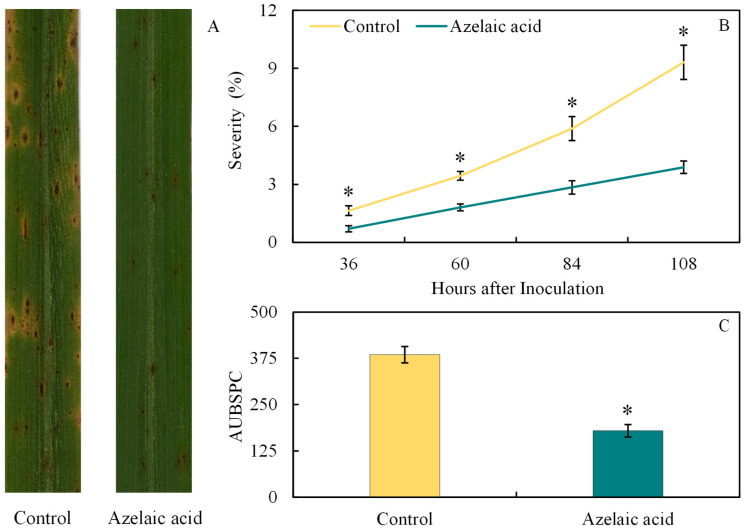
Disease symptoms, severity (**B**), and area under brown spot progress curve (AUBSPC) (**C**) for rice plants sprayed with water (control) or azelaic acid (10 mM). Images of leaves with brown symptoms were obtained at 108 h after inoculation with *Bipolaris oryzae* (**A**). Means for control and azelaic acid treatments followed by an asterisk (*) at each evaluation time (graph **B**) or between these treatments (graph **C**) are significantly different (*p* ≤ 0.05) according to the *F* test. Bars represent the standard error of the means.

The potential of AzA to reduce the development of brown spot in rice leaves was investigated by evaluating both disease symptoms and its progress as illustrated in Figure 1. As expected, lesions of reddish-brown color and an intense yellow halo showed coalescence on the leaves of plants sprayed with water in contrast to those formed on leaves sprayed with AzA (Figure 3A). Significant (*p* < 0.05; Table 1) reductions of 57, 48, 52, and 58% for disease severity at 36, 60, 84, and 108 hai, respectively, were obtained for AzA-sprayed plants compared to water-sprayed ones (Figure 3B). The AUBSPC significantly (*p* < 0.05; Table 1) decreased by 53% for AzA-sprayed plants compared to water-sprayed ones (Figure 3C).

### 3.3. Foliar Concentrations of MDA, *H_2_O_2,_ and* O_2_^•−^

**Figure 4:**
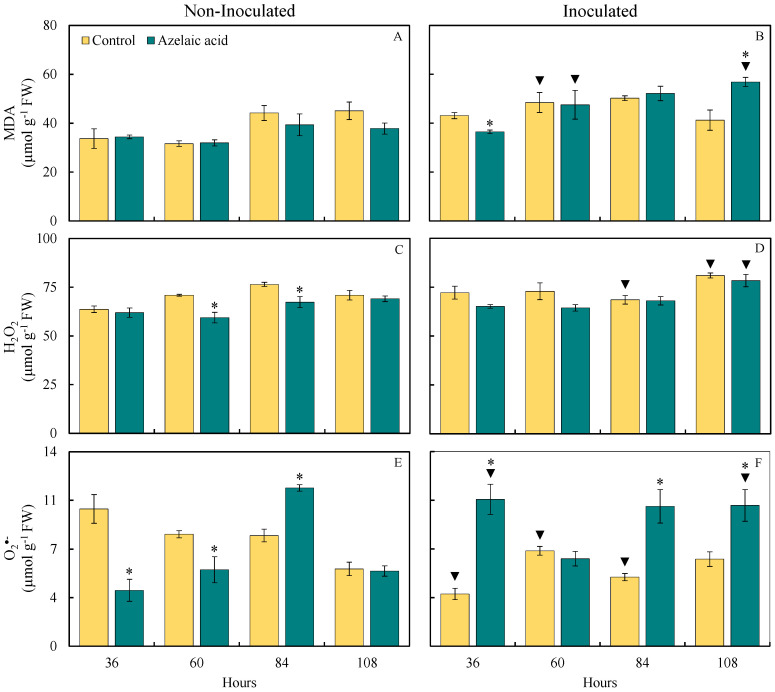
Foliar concentrations of malondialdehyde (MDA) (**A**,**B**), hydrogen peroxide (H_2_O_2_) (**C**,**D**), and superoxide anion radical (O_2_^•−^) (**E**,**F**) for rice plants sprayed with water (control) or azelaic acid (10 mM) and non-inoculated (**A**,**C**,**E**) or inoculated (**B**,**D**,**F**) with *Bipolaris oryzae*. For each evaluation time, means for non-inoculated and inoculated plants followed by an asterisk (*) and means for control and azelaic acid treatments followed by an inverted triangle (▼) are significantly different (*p* ≤ 0.05) according to the *F* test. Bars represent the standard error of the means. FW = fresh weight.

The effect of fungal infection on the oxidative stress in rice leaf tissues was investigated through the quantification of MDA, H_2_O_2,_ and O_2_^•−^ concentrations. The pool of these compounds provided insights into the level of damage caused by fungal infection in leaf tissues of plants sprayed with water in comparison to those sprayed with AzA (Figure 4). For plants non-challenged with the fungus, there were significant (*p* < 0.05; Table 1) reductions in the concentrations of H_2_O_2_ (16 and 12% at 60 and 84 hai, respectively) and O_2_^•−^ (62 and 32% at 36 and 60 hai, respectively) and a significant (*p* < 0.05; Table 1) increase of 48% for O_2_^•−^ concentration at 84 hai when they were sprayed with AzA compared to those from the control treatment (Figure 4C,E). For plants challenged with the fungus, the MDA concentration significantly (*p* < 0.05; Table 1) decreased by 15% at 36 hai while the concentrations of MDA (38% at 108 hai) and O_2_^•−^ (189, 103, and 72% at 36, 84, and 108 hai, respectively) significantly (*p* < 0.05; Table 1) increased when they were sprayed with AzA in contrast to those of the control treatment (Figure 4B,F).

For plants sprayed with water, there were significant (*p* < 0.05; Table 1) increases in the concentrations of MDA (53% at 60 hai), H_2_O_2_ (14% at 108 hai), and O_2_^•−^ (14% at 108 hai) and significant (*p* < 0.05; Table 1) reductions in the concentrations of H_2_O_2_ (10% at 84 hai) and O_2_^•−^ (15 and 38% at 60 and 84 hai, respectively) when they were challenged with the fungus compared to non-challenged ones (Figure 4A,D,F). For plants sprayed with AzA, there were significant (*p* < 0.05; Table 1) increases in the concentrations of MDA (49 and 50% at 60 and 108 hai, respectively), H_2_O_2_ (13% at 108 hai), and O_2_^•−^ (176 and 103% at 36 and 108 hai, respectively) when they were challenged with the fungus in contrast to non-challenged ones (Figure 4A,D,F).

### 3.4. Activities of Antioxidative Enzymes

**Figure 5:**
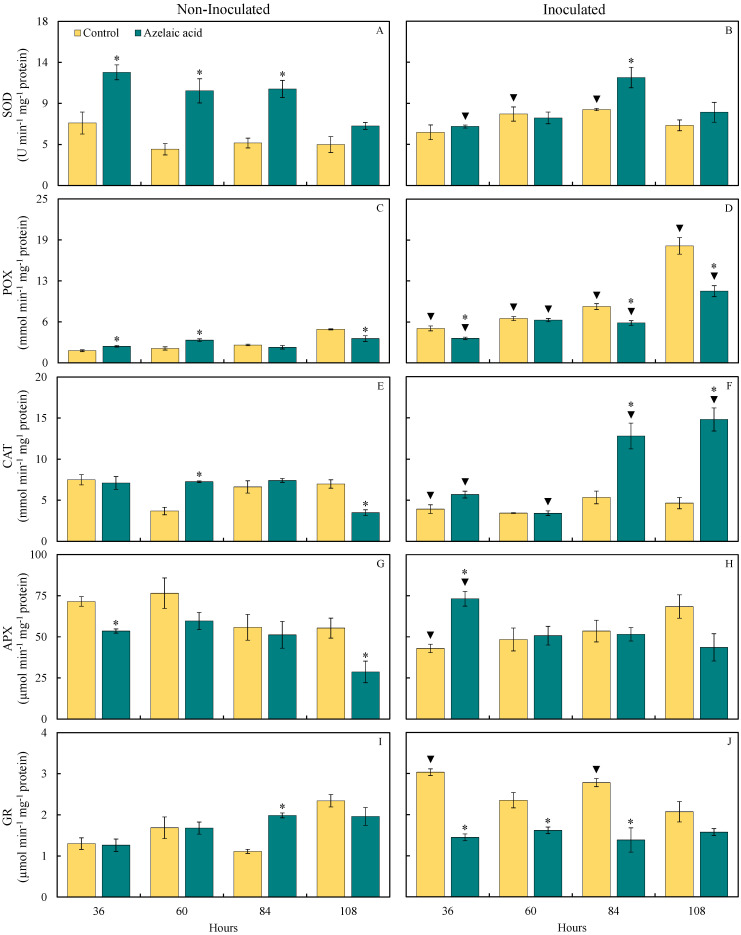
Activities of superoxide dismutase (SOD) (**A**,**B**), peroxidase (POX) (**C**,**D**), catalase (CAT) (**E**,**F**), ascorbate peroxidase (APX) (**G**,**H**), and glutathione reductase (GR) (**I**,**J**) determined on the leaves of rice plants sprayed with water (control) or azelaic acid (10 mM) and non-inoculated (**A**,**C**,**E**,**G**,**I**) or inoculated (**B**,**D**,**F**,**H**,**J**) with *Bipolaris oryzae*. For each evaluation time, means for non-inoculated and inoculated plants followed by an asterisk (*) and means for control and azelaic acid treatments followed by an inverted triangle (▼) are significantly different (*p* ≤ 0.05) according to the *F* test. Bars represent the standard error of the means.

The determination of activity for some key enzymes involved in the antioxidant metabolism was dictated by the level of response of rice plants sprayed with AzA in contrast to those that were water-sprayed following the challenge by *B. oryzae*. The pattern of SOD, POX, CAT, APX, and GR activities in leaves of rice plants sprayed with water or AzA and non-challenged or challenged with *B. oryzae* is shown in Figure 5. For non-challenged plants, there were significant (*p* < 0.05; Table 1) increases in SOD (81, 162, and 127% at 36, 60, and 84 hai, respectively), POX (37 and 58% at 36 and 60 hai, respectively), CAT (97% at 60 hai), and GR (79% at 84 hai) activities and significant (*p* < 0.05; Table 1) reductions in POX and CAT (28 and 50%, respectively, at 108 hai) as well as APX (25 and 48% at 36 and 108 hai, respectively) activities when they were sprayed with AzA compared to those from the control treatment (Figure 5A,C,E,G,I). For plants challenged with the fungus, there were significant (*p* < 0.05; Table 1) increases in SOD (42% at 84 hai), CAT (139 and 218% at 84 and 108 hai, respectively), and APX (70% at 36 hai) activities and significant (*p* < 0.05; Table 1) reductions in POX (29, 29, and 39% at 36, 84, and 108 hai, respectively) and GR (52, 31, and 50% at 36, 60, and 84 hai, respectively) activities after being sprayed with AzA in contrast to those from the control treatment (Figure 5B,D,F,H,J).

For plants sprayed with water, SOD (97 and 79% at 60 and 84 hai, respectively), POX (184, 208, 214, and 249% at 36, 60, 84, and 108 hai, respectively), and GR (133 and 150% at 36 and 84 hai, respectively) activities significantly (*p* < 0.05; Table 1) increased while CAT and APX activities significantly (*p* < 0.05; Table 1) decreased (48 and 40%, respectively, at 36 hai) when they were challenged with the fungus compared to non-challenged ones (Figure 5B,D,F,H,J). For plants sprayed with AzA, POX (48, 88, 157, and 196% at 36, 60, 84, and 108 hai, respectively), CAT (72 and 323% at 84 and 108 hai, respectively), and APX (37% at 36 hai) activities significantly (*p* < 0.05; Table 1) increased while SOD (48% at 36 hai) and CAT (53% at 60 hai) activities significantly (*p* < 0.05; Table 1) decreased when they were challenged with the fungus compared to non-challenged ones (Figure 5B,D,F,H).

### 3.5. Activities of Defense-Related Enzymes

**Figure 6:**
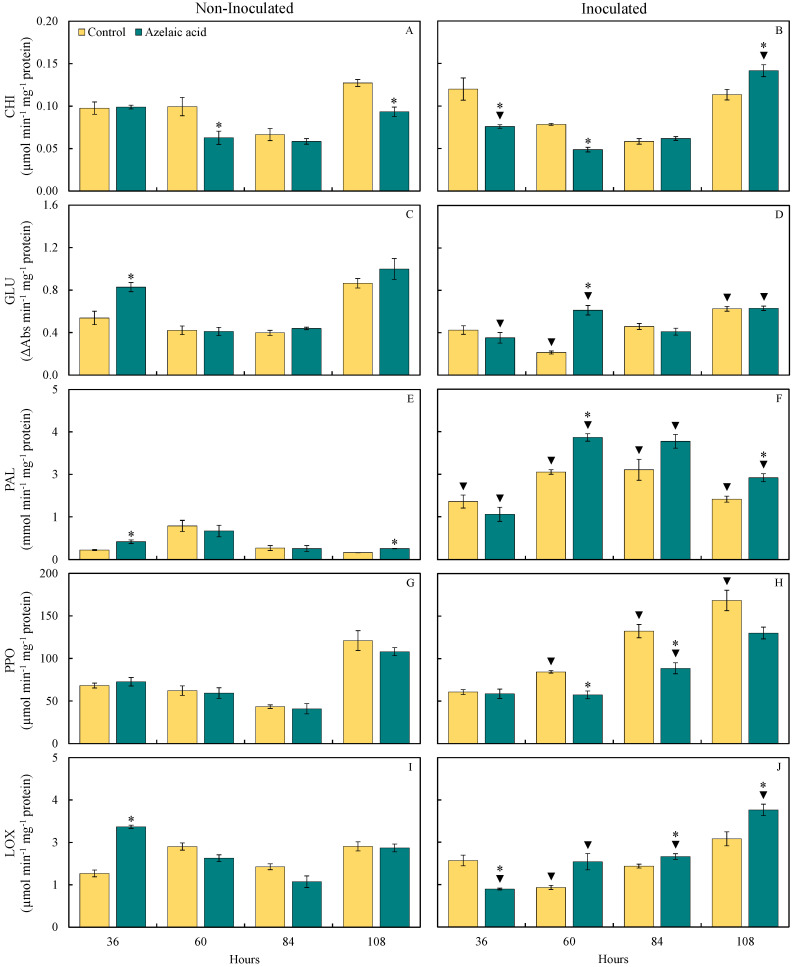
Activities of chitinase (CHI) (**A**,**B**), *β*-1,3-glucanase (GLU) (**C**,**D**), phenylalanine ammonia-lyase (PAL) (**E**,**F**), polyphenoloxidase (PPO) (**G**,**H**), and lipoxygenase (LOX) (**I**,**J**) determined on the leaves of rice plants sprayed with water (control) or azelaic acid (10 mM) and non-inoculated (**A**,**C**,**E**,**G**,**I**) or inoculated (**B**,**D**,**F**,**H**,**J**) with *Bipolaris oryzae*. For each evaluation time, means for non-inoculated and inoculated plants followed by an asterisk (*) and means for control and azelaic acid treatments followed by an inverted triangle (▼) are significantly different (*p* ≤ 0.05) according to the *F* test. Bars represent the standard error of the means.

The status of enzyme activities related to host defense such as CHI, GLU, PAL, PPO, and LOX in plant tissues infected by *B. oryzae* was assessed for the water- and AzA-sprayed plants in terms of brown spot control (Figure 6). For non-challenged plants, GLU and LOX (54 and 87%, respectively, at 36 hai) as well as PAL (88 and 57% at 36 and 108 hai, respectively) activities significantly (*p* < 0.05; Table 1) increased while CHI activity significantly (*p* < 0.05; Table 1) decreased (37 and 27% at 60 and 84 hai, respectively) when they were sprayed with AzA compared to those from the control treatment (Figure 6A,C,E,I). For plants challenged with the fungus, CHI (25% at 108 hai), GLU (186% at 60 hai), PAL (40 and 35% at 60 and 108 hai, respectively), and LOX (16 and 33% at 84 and 108 hai, respectively) activities significantly (*p* < 0.05; Table 1) increased when they were sprayed with AzA compared to those from the control treatment (Figure 6B,D,F,J). Activities of CHI (37 and 38% at 36 and 60 hai, respectively), PPO (32 and 33% at 60 and 84 hai, respectively), and LOX (43% at 36 hai) activities significantly (*p* < 0.05; Table 1) decreased for AzA-sprayed challenged plants compared to water-sprayed challenged ones (Figure 6B,H,J).

For plants sprayed with water, PAL (507, 159, 675, and 760% at 36, 60, 84, and 108 hai, respectively) and PPO (36, 205, and 39% at 60, 84, and 108 hai, respectively) activities significantly (*p* < 0.05; Table 1) increased while GLU (49 and 28% at 60 and 108 hai, respectively) and LOX (51% at 60 hai) activities significantly (*p* < 0.05; Table 1) increased for when they were challenged with the fungus compared to non-challenged ones (Figure 6B,D,H,J). For plants sprayed with AzA, GLU (49% at 60 hai), PAL (152, 326, 967, and 640% at 36, 60, 84, and 108 hai, respectively), CHI (51% at 108 hai), PPO (117% at 84 hai), and LOX (55 and 48% at 84 and 108 hai, respectively) activities significantly (*p* < 0.05; Table 1) increased while GLU (57 and 37% at 36 and 108 hai, respectively) as well as CHI and LOX (23 and 62%, respectively, at 36 hai) activities significantly (*p* < 0.05; Table 1) decreased when they were challenged with the fungus compared to non-challenged ones (Figure 6B,D,F,H,I,J).

### 3.6. Foliar Concentrations of TSP and LTGA Derivatives

**Figure 7:**
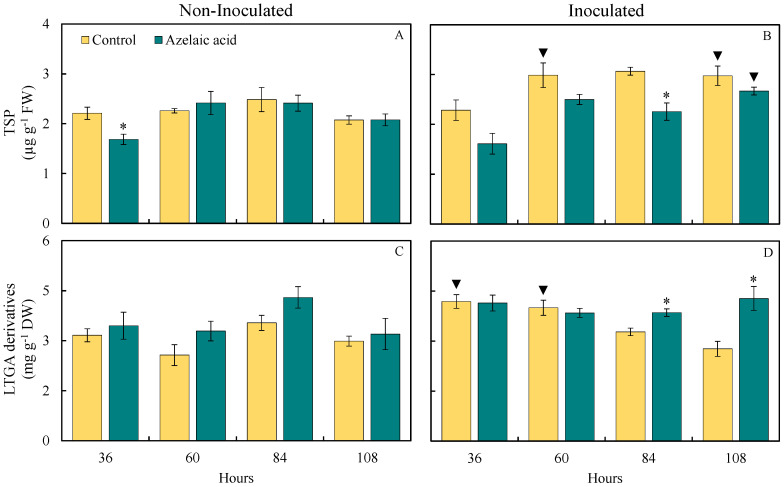
Foliar concentrations of total soluble phenolics (TSP) (**A**,**B**) and lignin-thioglycolic acid (LTGA) derivatives (**C**,**D**) for rice plants sprayed with water (control) or azelaic acid (10 mM) and non-inoculated (**A**,**C**) or inoculated (**B**,**D**) with *Bipolaris oryzae*. For each evaluation time, means for non-inoculated and inoculated plants followed by an asterisk (*) and means for control and azelaic acid treatments followed by an inverted triangle (▼) are significantly different (*p* ≤ 0.05) according to the *F* test. Bars represent the standard error of the means. FW and DW = fresh weight and dry weight, respectively.

The quantification of the compound TSP and LTGA derivatives, which are intrinsically associated with the level of resistance of plants against pathogens, was determined by the response of rice plants sprayed with AzA against fungal infection (Figure 7). Interestingly, TSP concentration was significantly (*p* < 0.05; Table 1) lower (17% at 36 hai) and the concentration of LTGA derivatives significantly (*p* < 0.05; Table 1) increased (21, 24, 27, and 32% at 36, 60, 84, and 108 hai, respectively) for plants sprayed with AzA compared to those from the control treatment in the absence of fungal infection (Figure 7A,C). For plants challenged with the fungus, the TSP concentration was significantly (*p* < 0.05; Table 1) lower (26% at 84 hai), while the concentration of LTGA derivatives significantly (*p* < 0.05; Table 1) increased (18 and 55% at 84 and 108 hai, respectively) when they were sprayed with AzA in contrast to water-sprayed ones (Figure 7B,D).

For plants from the control treatment, there were significant (*p* < 0.05; Table 1) increases in the concentrations of TSP (32, 37, and 43% at 60, 84, and 108 hai, respectively) and LTGA derivatives (32 and 33% at 36 and 60 hai, respectively) when they were challenged with *B. oryzae* compared to non-challenged ones (Figure 7B,D). For plants from the control treatment, there were significant (*p* < 0.05; Table 1) increases in the concentrations of TSP and LTGA derivatives of 28% at 108 hai and 21% at 36 hai, respectively, when they were challenged with *B. oryzae* in contrast to non-challenged ones (Figure 7B,D).

### 3.7. PCA

**Figure 8:**
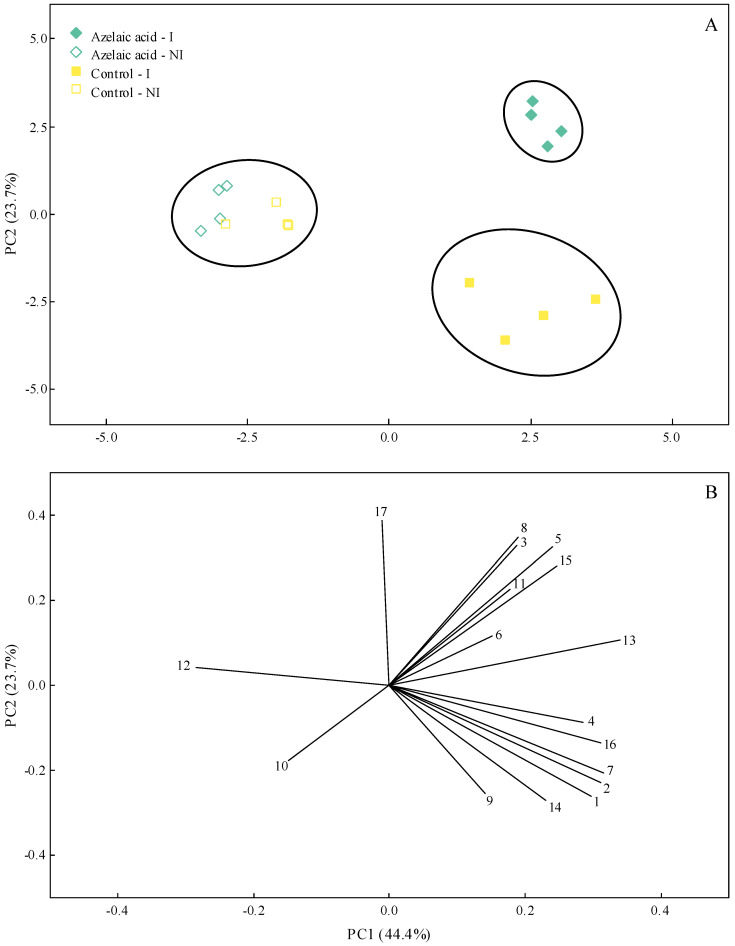
Principal component analysis (PCA) showing the score plots (**A**) and loading values (**B**) for disease and biochemical variables evaluated in rice plants sprayed with water (control) or azelaic acid (10 mM) and non-inoculated (NI) or inoculated (I) with *Bipolaris oryzae*. The numbers in the PCA are as follows: disease severity (1), area under brown spot progress curve (2), compounds (3, 4, and 5, respectively, refer to malondialdehyde, hydrogen peroxide, and superoxide anion radical) and activities of antioxidant (6, 7, 8, 9, and 10, respectively, refer to superoxide dismutase, peroxidase, catalase, ascorbate peroxidase, and glutathione reductase) and defense-related (11, 12, 13, 14, and 15, respectively, refer to chitinase, *β*-1,3-glucanase, phenylalanine ammonia-lyase, polyphenoloxidase, and lipoxygenase) enzymes, and metabolites (16 and 17, respectively, refer to total soluble phenolics and lignin-thioglycolic acid derivatives). Groups were generated from cluster analysis with complete linkage and Pearson distance. Data from variables used in the PCA were obtained at 108 h after inoculation for inoculated plants and for non-inoculated plants at this same time-point.

In order to capture most of the biological variation in the variables and parameters evaluated in this study according to the four treatments used (plants non-challenged or challenged with *B. oryzae* that were exposed to water or AzA), a PCA was carried out. The information gained using this type of analysis highlighted the potential of AzA to reduce disease symptoms; at the same time, this molecule guaranteed a better physiological and biochemical response of infected plants. Interestingly, most of the data variation was explained by one principal component (PC) (PC1 = 44.4% and PC2 = 23.7%) (Figure 8A,B). According to the cluster analysis with complete linkage and Pearson distances, two clusters were formed for inoculated plants from the control and AzA treatments, while one independent cluster originated from the non-inoculated plants from the control and AzA treatments (Figure 8A). PC1 resulted in negative scores for GR, GLU, and LTGA derivatives and positive scores for the other variables and parameters evaluated (Figure 8B). Negative scores for Sev, AUBSPC, H_2_O_2_, POX, APX, GR, PPO, and TSP and positive scores for the other variables and parameters evaluated were obtained for PC2 (Figure 8B).

## 4. Discussion

Resistance inducers are a sustainable approach to protect profitable crops against pathogens causing destructive diseases, considering that defense responses originating from different metabolic pathways will be rapidly and efficiently boosted. The present study reports the potential of AzA to reduce the severity of brown spot on leaves of rice plants based on the outcome obtained from the physiological and biochemical data. Interestingly, the AzA negatively affected conidium germination and fungal mycelial growth in vitro. It is plausible to speculate that the AzA may cause osmotic stress, ion imbalance, and changes in membrane integrity of germ tubes and mycelia. Studies reporting the effect of AzA inhibiting the physiology of pathogens are scarce in the literature. For instance, the AzA showed a direct antifungal activity against the germination of urediniospores from *Phakopsora pachyrhizi* and *Hemileia vastatrix* in vitro [14,15].

Plants have developed efficient mechanisms of biochemical defense against infection by pathogens, such as the action of different pathogenesis-related proteins [30]. In the present study, the CHI and GLU activities were greater at 108 hai and 60 ha, respectively, for diseased plants exposed to AzA in contrast to plants that were not exposed to AzA but infected by *B. oryzae*. This finding indicates a role of AzA in reducing the colonization of rice leaf tissues by *B. oryzae,* considering the degradation of the fungal cell wall by the enzymes CHI and GLU. PAL is an important enzyme involved in the phenylpropanoid pathway from which several secondary metabolites, such as phenolics, flavonoids, and phytoalexins, are formed [4]. Lignin formation is PAL-dependent and acts in the strength of the cell wall [31]. In the present study, more precisely at 108 hai, PAL activity was higher for diseased plants that received AzA, resulting in a greater concentration of LTGA derivatives. There was greater PAL activity and, consequently, greater accumulation of LTGA derivatives in soybean plants infected with *P. pachyrhizi* and treated with AzA compared with infected plants sprayed with water [15]. PPO is also an important enzyme involved in the stress response of plants infected by pathogens due to its participation in forming phenolics in plant tissues [32]. Interestingly, PPO activity was not higher in the leaf tissues of diseased plants that were previously pulverized with AzA, which was hypothesized to result in a greater pool of phenolics. LOX is an enzyme that catalyzes the oxidation of fatty acids to form linoleic acid, which is subsequently used to form jasmonic acid, which is involved in the induced systemic resistance against pathogens [33]. In the present study, LOX activity was greater for diseased plants that received AzA in contrast to diseased plants that received water spray at 60 and 108 hai. It is worth mentioning that LOX activity was significantly higher for non-infected plants that received AzA at 36 hai compared to non-infected plants sprayed with water. In the present study, earlier activities of GLU, PAL, and LOX for non-infected plants pulverized with AzA indicated the potential of this molecule to prime the leaf tissues in the absence of fungal infection. Interestingly, activities of CHI, GLU, PAL, POX, and PPO were lower for coffee plants sprayed with AzA as a result of reduced colonization and sporulation by *H. vastatrix* in contrast to plants that received water and were infected by this fungus [15]. On the other hand, the reduced severity of rust on coffee leaves was attributed to higher concentration of O_2_^•−^ for plants that received AzA at earlier stages of infection by *H. vastatrix* that hampered the colonization of leaf tissues by fungal hyphae and affected haustoria formation, as well as having a citotoxic fungistatic effect against the fungus [15]. The resistance of tomato plants against infection by *Alternaria solani* increases after foliar spray with AzA [34]. According to the authors, the activities of CHI, laccase, PAL, and LOX, as well as the concentrations of phenolics and flavonoids, were higher in the plants that received AzA during the fungal infection process. Increased lignin deposition in infected leaf tissues slowed fungal colonization, reducing early blight severity [34].

Plants exposed to various types of stress, whether abiotic or biotic, develop an enzymatic strategy to control the concentrations of reactive oxygen species, in which SOD, CAT, APX, POX, and GR play pivotal roles [35]. APX is one of the main enzymes in the antioxidant system that catalyzes the breakdown of H_2_O_2_ into water and oxygen using the ascorbate as an electron donor [36]. The present study observed an increase in APX activity at 36 hai for diseased plants pulverized with AzA compared to diseased plants that received water. MDA is a metabolite that indicates a higher level of cellular lipid peroxidation [37]. Considering the lower concentration of MDA at 36 hai, it is plausible to postulate that APX helped to lower the pool of ROS and, consequently, the level of leaf tissue degradation during the infection of *B. oryzae*. SOD acts to remove O_2_^•−^ from the leaf tissues to form H_2_O_2_ [38]. On the other hand, CAT catalyzes the decomposition of hydrogen peroxide into water and molecular oxygen, thereby reducing its harmful effects on plant tissues [39]. In the present study, non-infected plants that received AzA displayed increased SOD activity and consequently a reduced pool of O_2_^•−^ at 60 hai as well as an increase in CAT activity and reduced concentration of H_2_O_2_. A reduction in SOD activity at 36 hai was also observed for diseased plants that received AzA compared to water-sprayed and infected ones, resulting, therefore, in an increase in the concentration of O_2_^•−^. POX is also important to alleviate the stress in the plant leaf tissues imposed by pathogen infections [4]. In the present study, non-diseased plants that were sprayed with AzA showed greater POX activity at 60 hai, resulting in a lower concentration of H_2_O_2_ than diseased plants sprayed with water. In the present study, greater GR activity and a reduction in the pool of H_2_O_2_ were obtained for non-diseased plants pulverized with AzA at 84 hai. O_2_^•−^ is a free radical produced in plant tissues facing infection by pathogens and may act as a cellular signal to boost cell defense reactions [4]. In the present study, the highest concentrations of O_2_^•−^ and LTGA derivatives were observed at 84 and 108 hai for diseased plants that received AzA in contrast to diseased plants previously exposed to water. Coffee and soybean plants sprayed with AzA and infected by *H. vastatrix* and *P. pachyrhizi*, respectively, displayed a more robust antioxidative metabolism as a result of greater activities of APX, CAT, GR, and POX, and this helped to reduce the pool of H_2_O_2_ and O_2_^•−^ in the infected leaf tissues [15,40]. In general, the activities of enzymes involved in alleviating the oxidative stress were affected according to the treatments imposed on plants. For example, non-diseased plants that received AzA showed a better antioxidative metabolism. On the contrary, the pattern for POX and GR activities changed in contrast to APX, CAT, and SOD activities for diseased plants that received this same molecule. These changes were of pivotal importance to hamper the infection process of *B. oryzae*.

Taken together, the AzA is shown to be an interesting signaling molecule capable of modulating the biochemical responses (e.g., greatest activities of CAT, POX, GR, and SOD linked to lower pools of H_2_O_2_ and O_2_^•−^ as well as higher activities of GLU, PAL, and LOX) of non-infected plants. Upon fungal infection, primed rice plants mounted a more efficient biochemical defense response, resulting in reduced disease symptoms. In addition, the AzA-sprayed plants exhibited a reasonably efficient antioxidant metabolism, alleviating the cellular stress imposed by fungal infection. Interestingly, based on the PCA, it was possible to clearly separate the outcome of AzA- and water-sprayed plants facing fungal infection for the variables and parameters studied. Future studies that cover transcriptomic, proteomic, metabolomic, and functional genetic approaches in rice plants exposed to AzA and infected with *B. oryzae* will help us better understand the role of this molecule in boosting plant resistance and reducing disease severity. It is plausible to conclude that the use of AzA, together with other available control strategies, may be outstandingly advantageous for managing brown spot while heading towards more sustainable agriculture.

## Figures and Tables

**Table 1:** Analysis of variance to determine the effects of product (P), plant inoculation (PI), and the interaction P × PI, at each evaluation time, on the area under brown spot progress curve (AUBSPC), brown spot severity (Sev), concentrations of metabolites [malondialdehyde (MDA), hydrogen peroxide (H_2_O_2_), and superoxide anion radical (O_2_^•−^)], activities of antioxidant enzymes [superoxide dismutase (SOD), peroxidase (POX), catalase (CAT), ascorbate peroxidase (APX), and glutathione reductase (GR)], and activities of defense-related enzymes [chitinase (CHI), *β*-1,3-glucanase (GLU), phenylalanine ammonia-lyase (PAL), polyphenoloxidase (PPO), and lipoxygenase (LOX)] as well as concentrations of total soluble phenolics (TSP) and lignin-thioglycolic acid (LTGA) derivatives.

Variables/Parameters	36 hai			60 hai			84 hai			108 hai		
	P	PI	P × PI	P	PI	P × PI	P	PI	P × PI	P	PI	P × PI
AUBSPC	**<0.001**	**-**	-	**-**	**-**	-	**-**	**-**	-	**-**	**-**	-
Sev	**0.019**	**-**	-	**0.001**	**-**	-	**0.005**	**-**	-	**0.001**	**-**	-
MDA	0.198	**0.022**	0.119	0.936	**0.001**	0.866	0.652	**0.011**	0.299	0.204	**0.031**	**0.003**
H_2_O_2_	0.075	**0.022**	0.261	**0.003**	0.219	0.588	**0.045**	0.118	0.070	0.340	**0.001**	0.864
O_2_^•−^	0.621	0.782	**<0.001**	**0.018**	0.760	0.082	**<0.001**	**0.004**	0.338	**0.003**	**<0.001**	**0.001**
SOD	**0.008**	**0.004**	**0.026**	**0.013**	0.681	**0.006**	**<0.001**	**0.019**	0.204	0.077	0.071	0.741
POX	0.134	**<0.001**	**0.001**	0.109	**<0.001**	**0.026**	**0.002**	**<0.001**	**0.013**	**0.001**	**<0.001**	**0.011**
CAT	0.333	**0.004**	0.144	**<0.001**	**<0.001**	**<0.001**	**0.003**	0.091	**0.011**	**0.005**	**0.001**	**<0.001**
APX	0.107	0.228	**<0.001**	0.382	**0.040**	0.255	0.688	0.907	0.873	**0.008**	0.110	0.912
GR	**<0.001**	**<0.001**	**<0.001**	0.101	0.175	0.108	0.185	**0.014**	**<0.001**	0.063	0.151	0.795
CHI	**0.016**	0.953	**0.012**	**<0.001**	**0.024**	0.611	0.609	0.589	0.217	0.626	**0.012**	**<0.001**
GLU	0.082	**<0.001**	**0.008**	**0.001**	0.950	**<0.001**	0.893	0.582	0.148	0.301	**0.001**	0.333
PAL	0.709	**<0.001**	0.084	**0.016**	**<0.001**	**0.003**	0.088	**<0.001**	0.078	**0.001**	**<0.001**	**0.011**
PPO	0.805	**0.048**	0.522	**0.020**	0.094	**0.049**	**0.006**	**<0.001**	**0.011**	**0.035**	**0.007**	0.266
LOX	**0.033**	**<0.001**	**<0.001**	0.236	**0.002**	**0.006**	0.545	**0.011**	**0.013**	**0.050**	**0.004**	**0.032**
TSP	**0.007**	0.228	0.581	0.371	**0.042**	0.097	**0.025**	**0.021**	**0.002**	0.256	**<0.001**	0.250
LTGA derivatives	**0.019**	**0.001**	0.580	0.087	**0.003**	**0.014**	**0.001**	**0.033**	0.327	**<0.001**	0.811	0.272

Bold values are significant at *p* < 0.05.

## Data Availability

The raw data supporting the conclusions of this article will be made available by the authors on request.
